# Female and Male Moths Display Different Reproductive Behavior when Facing New versus Previous Mates

**DOI:** 10.1371/journal.pone.0109564

**Published:** 2014-10-07

**Authors:** Yan-Ying Li, Jin-Feng Yu, Qin Lu, Jin Xu, Hui Ye

**Affiliations:** 1 Yunnan key laboratory of international rivers and transboundary eco-security, Yunnan University, Kunming, P.R. China; 2 Key laboratory for animal genetic diversity and evolution of high education in Yunnan province, Yunnan University, Kunming, P.R. China; CNRS, France

## Abstract

Multiple mating allows females to obtain material (more sperm and nutrient) and/or genetic benefits. The genetic benefit models require sperm from different males to fertilize eggs competitively or the offspring be fathered by multiple males. To maximize genetic benefits from multiple mating, females have evolved strategies to prefer novel versus previous mates in their subsequent matings. However, the reproductive behavior during mate encounter, mate choice and egg laying in relation to discrimination and preference between sexes has been largely neglected. In the present study, we used novel and previous mate treatments and studied male and female behavior and reproductive output in *Spodoptera litura*. The results of this study do not support the sperm and nutrient replenishment hypotheses because neither the number of mates nor the number of copulations achieved by females significantly increased female fecundity, fertility and longevity. However, females showed different oviposition patterns when facing new versus previous mates by slowing down oviposition, which allows the last male has opportunities to fertilize her eggs and the female to promote offspring diversity. Moreover, females that have novel males present called earlier and more than females that have their previous mates present, whereas no significant differences were found on male courtship between treatments. These results suggest that *S. litura* females can distinguish novel from previous mates and prefer the former, whereas males generally remate regardless of whether the female is a previous mate or not. In *S. litura*, eggs are laid in large clusters and offspring competition, inbreeding and disease transfer risks are thus increased. Therefore, offspring diversity should be valuable for *S. litura*, and genetic benefits should be the main force behind the evolution of female behavioral strategies found in the present study.

## Introduction

Mating is very costly due to energy consumption, the risks of disease transmission and predation, and injury caused during mating [1], [2]. Both theoretical [3] and empirical (e.g., [4], [5]) studies have demonstrated that one copulation is adequate for females to obtain their maximum reproductive success in many insect species. However, the majority of female insects prefer to mate multiply, not only with different males (multiple mating) but also with the same male (repeated mating) [1].

Two categories of hypotheses have been developed to explain the evolutionary significance of female multiple mating: to obtain material benefits and to gain genetic benefits. Females may obtain material benefits from multiple mating by one or more of the following ways [1], [6], [7]: 1) sperm replenishment – females remate to obtain adequate sperm to ensure her full load of eggs can be fertilized; 2) nutrient replenishment – females remate to obtain male invested nutrients for longer longevity or higher fecundity; and 3) convenience benefits – females remate to minimize the cost from male harassment.

A number of genetic benefits hypotheses have also been suggested to explain the evolutionary significance of female multiple mating: 1) good genes – paternally derived genes enhanced the attractiveness of offspring or genes from their parents improved the survival of offspring [8], [9]; 2) genetic incompatibility – females can bias paternity and give priority to males with good or more compatible genes to fertilize her eggs [10]–[13]; 3) genetic diversity – by increasing genetic diversity within progeny, females guard against future environmental uncertainty [14], [15], offspring benefit from enhanced genetic diversity by disease resistance, niche separation [16], [17] and inbreeding avoidance [4], [18].

To maximize the genetic benefits from multiple mating, females have evolved strategies to prefer novel versus previous mates in their subsequent matings [19]. Thus far, nine studies have investigated whether females can discriminate between novel and previous mates in their subsequent matings and found that it is positive in five invertebrate [4], [19]–[22] and two vertebrate [23], [24] species and negative only in two invertebrate species [25], [26]. Newcomer *et al.* [27] found that in the pseudoscorpion *Cordylochernes scorpioides,* females increased offspring viability by discriminating against previous mates. Archer & Elgar [21] and Xu & Wang [4] demonstrate that females of the hide beetle *Dermestes maculatus* and the Mediterranean flour moth *Ephestia kuehniella* choose new mates for remating to gain genetic benefits from increased offspring diversity. These two studies [4], [21] also found that a female might delay or slow down oviposition if has a novel male around after her first mating compared to a female still caged with her previous mate after her first mating.

Compared to females, it is generally accepted that multiple mating can significantly improve males’ reproductive success [28], [29]. Studies also found that males of many taxa, including insects, displayed a heightened preference for novel mates combined with a decline in the propensity to remating with the same mate [26]. However, two other studies in insects have found the opposite result: males significantly prefer previous to new mates for subsequent matings [19], [30]. Moreover, very few studies have tested the preference for novel mates in both males and females [26], making the reproductive behavior during mate encounter, mate choice and egg laying in relation to discrimination and preference between sexes be largely neglected.

The tobacco cutworm, *Spodoptera litura* (Fabricius, 1775) (Lepidoptera: Noctuidae), is one of the most serious agricultural pests worldwide [31]. This insect is a nocturnal moth and all adult sexual activities (courtship, calling, mating and oviposition) take place during the scotophase [32]. Adult moths eclose at dusk, and no matings take place during the night of eclosion. Approximately 70% of mating occurs on the subsequent night after emergence, and those unmated will mate on the third night [32], [33]. Paired insects can mate up to four times, with an average of 1.9±0.4 matings [32]. Females begin to lay eggs on the subsequent night after first mating [32], [33].

In the present study, we allow *S. litura* females and males to mate the first time during the second night after eclosion and then allow them to encounter novel or previous mates on the subsequent night to test female and male responses to mate-novelty. We also conducted single mating, repeated mating and multiple mating treatments to test the effect of the number of matings and mates on fecundity, fertility, oviposition pattern and longevity. Based on the behavioral and reproductive output test, we discussed the evolutionary significance of the behavioral strategies used by females when facing new versus previous mates.

## Materials and Methods

### Insects

*Spodoptera litura* were reared under a 14∶10 h light:dark photoperiod regime, at 26°C and 60–80% relative humidity. Larvae were fed on an artificial diet [34]; adults were reared on a 10% honey solution. This moth was found in Liujia Village, Kunming City of China in July 2012. The present study was conducted in May 2013. The insect has eight generations been in the lab before this study.

Pupae were sexed based on the morphology of exterior parameres [35]. Male and female pupae were caged separately and allowed to emerge to ensure virginity. Newly emerged moths (<12 h old) were collected from the colony and weighed to an accuracy of 0.0001 g using an electronic balance (Sartorius Bp221S, Germany). Mean body weight (mean ± SD) was 148.9±17.3 mg and 189.3±22.8 mg for male and female moths, respectively. Only moths with an average body weight (within one SD of the mean) [33] were used in this study.

### Effect of multiple mating on female reproductive behavior and success

On the second night after eclosion, male and female virgin moths were paired in plastic boxes (25 cm long, 15 cm wide, 8 cm high) for the whole night for mating, using one pair per box (n = 150). Females and males were seperated immediately after mating and were reared individually in new boxes (no oviposition was found on this night). At the beginning of the subsequent night (i.e., the third night after eclosion), the one-time mated females and males were used for further tests following different treatments in new boxes: (1) a female was individually caged for the remainder of her lifespan (1 male 1 mating) (n = 13), (2) a female was re-caged with her previous mate for the remainder of their lifespan (Paired) (n = 12), this treatment allows multiple matings to the same male, (3) a female was caged with a novel male for this night (this male had mated once on the previous night with another female) (Novel) (n = 12), (4) a female was caged with a novel male for this night (this male had mated once on the previous night with another female), unmated females in this night were collected and individually caged for the remainder of their lifespan (2 males 1 mating) (n = 12), (5) a female was caged with a novel male for this night (this male had mated once on the previous night with another female), mated females in this night were collected and individually caged for the remainder of their lifespan (2 males 2 matings) (n = 12), and (6) a female was caged with a virgin male by one virgin male per night until death (Multiple) (n = 12). All treatments were conducted on a day-night reversed cycle under above mentioned condition. Each box provided 10% honey solution as food, and a paper strip (15×20 cm) folded in zig-zag fashion was used as an oviposition substratum. Boxes were changed daily for all replications. A 15 W red light was used for illumination during observation.

To test the effect of mate-novelty on behavior in both males and females, the female calling, male courtship and mating during the third night after eclosion (the subsequent night after first mating, one-time mated females were exposed to previous or novel mates on this night) in treatments of Paired (n = 12) and Novel (n = 12) were recorded. The following behaviors were recorded by quickly observing all treatment moths every 10 min: calling – the pheromone gland was expanded and extruded [36]; courtship – the male jumped and fanned his wings around or over the female or if the male exposed his genitalia trying to engage the female’s genitalia; mating – the two moths engaged by the genitalia.

To study the lifelong remating and multiple mating patterns of this insect, the mating events in treatments of Paired and Multiple were recorded daily (all matings occurred in the night and the mating duration (mean ± SD) is 44.1±6.1 min for this insect, and thus matings were recorded by quickly observing treated insects every 30 min [32]).

To test the effect of multiple mating on female fecundity and fertility, both daily and total numbers of eggs laid were counted for each female moth of all treatments except treatment Novel. Females of Novel may mate once or twice during the treatments and thus we designed two other treatments (2 males 1 mating and 2 males 2 matings) as mentioned above to test the effect of the mate-novelty and multiple mating on female oviposition pattern and fecundity independently. Eggs were collected daily (females mated the first time on the second night after eclosion and started to lay eggs on the subsequent night) and incubated in Petri dishes (8.5×1.5 cm) under above mentioned conditions. Eggs having black dots (larval heads) after 2 days of incubation were recorded as fertilized [32]. The number of hatched eggs was recorded 4 days after incubation. Female longevity was also recorded. Dead females were dissected to count the number of spermatophores in their bursa copulatrix under a dissecting microscope.

### Statistics

Data on pre-calling duration (the duration between the start of the night and the start of calling), pre-mating duration (the duration between the start of the night and the start of mating), calling duration, hourly percentages of calling females and courting males on the subsequent night after first mating (one-time mated females were exposed to previous or novel mates on this night) were analyzed using ANOVA. Data on percentages of calling females or courting males were arcsin square root transformed. Data on hourly percentages of mated pairs on the subsequent night after first mating were not normally distributed after various transformations and thus were analyzed using a nonparametric Kruskal-Wallis test.

A Pearson chi-squared test was used to analyze the data from mating frequencies. Data on the overall number of eggs laid (fecundity), number of fertilized eggs laid (fertility), fertility rate (number of fertilized eggs laid/number of eggs laid), number of hatched eggs, hatch rate (number of hatched eggs/number of eggs laid) and female longevity were analyzed using a multivariate analysis of variance (MANOVA) as these data were collected from a repeated-measures and they might correlated each other [37]. MANOVA allows effects on both overall dependent variables and each dependent variable to be tested [37]. Prior to MANOVA, data on fertility rate and hatch rate were arcsin square root transformed.

Data on daily eggs laid were analyzed using an ANOVA followed by Fisher’s LSD test. The rejection level was set at *α*<0.05. All analyses were performed using SAS 9.1 [38]. Unless stated otherwise, all values were reported as mean ± SE.

## Results

**Figure 1:**
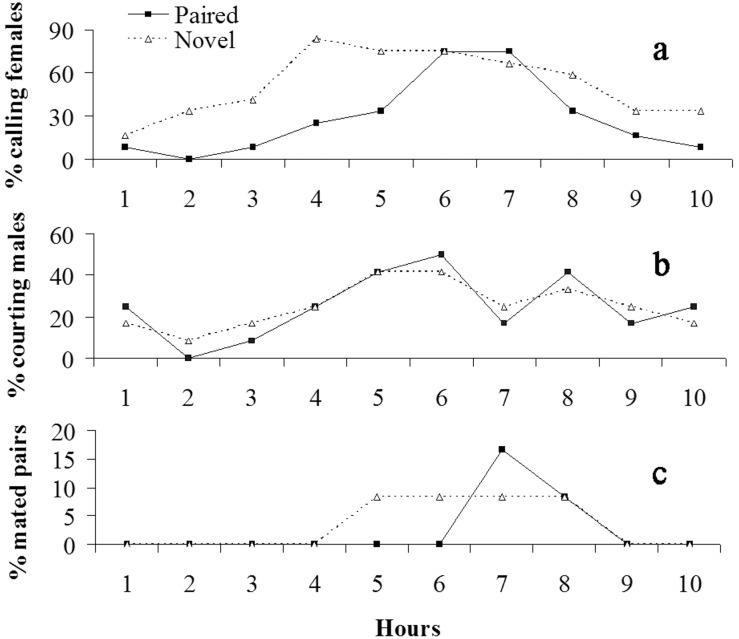
Hourly percentages of calling females (a), courting males (b) and mated pairs (c) in Novel and Paired treatments in *S. litura*.

Behavioral data on the subsequent night after first mating (one-time mated females were exposed to previous or novel mates on this night) revealed that females called at a higher rate when novel males were present (Novel) than that of females when previous mates were present (Paired) (ANOVA: *F*_1,18_ = 4.89, *P* = 0.040) (Fig. 1a). Further analysis also showed Novel females have significantly shorter pre-calling duration (309±24 min for Paired and 160±29 min for Novel; ANOVA: *F*_1,22_ = 7.08, *P* = 0.011) but longer calling duration (115±14 min for Paired and 217±18 min for Novel; ANOVA: *F*_1,22_ = 18.97, *P*<0.0001) than Paired females. However, no significant difference was found on hourly percentages of courting males between Paired and Novel treatments (ANOVA: *F*_1,18_ = 0.09, *P* = 0.767) (Fig. 1b).

**Figure 2:**
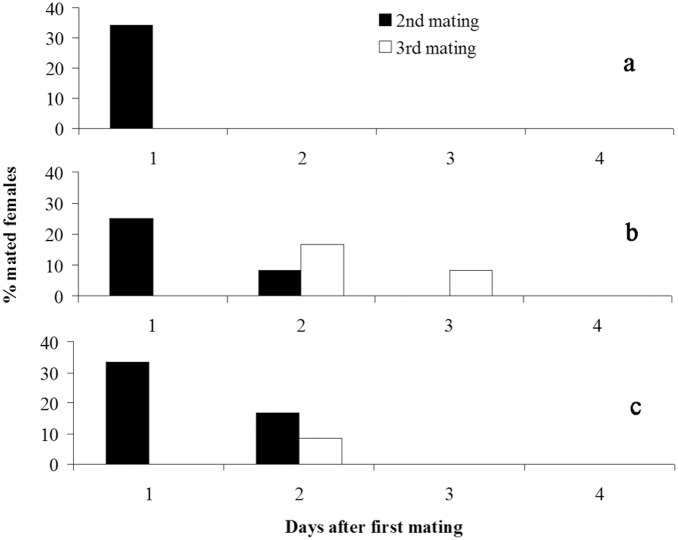
Remating patterns of females in treatment Novel (a), Paired (b) and Multiple (c) treatments in *S. litura*.

Approximately 30% of females mated the second time with the previous or novel males on the subsequent night after first mating (Fig. 1c; Fig. 2), and several females (<20%) mated a second and third time on following days in Paired and Multiple treatments (Figs. 2b & c). There was no significant difference on hourly percentages of mated pairs (Kruskal-Wallis test: *H*_1,18_ = 0.57, *P* = 0.451) and pre-mating durations (ANOVA: *F*_1,5_ = 0.05, *P* = 0.829) between treatment Paired and Novel (Fig. 1c). There was also no significant difference in mating numbers on the subsequent night after first mating between treatments of Novel, Paired and Multiple (Pearson chi-squared test: *DF* = 2, *χ^2^* = 0.363, *P* = 0.834) (Fig. 2). The number of spermatophores in females was equal to the number of matings observed.

**Figure 3:**
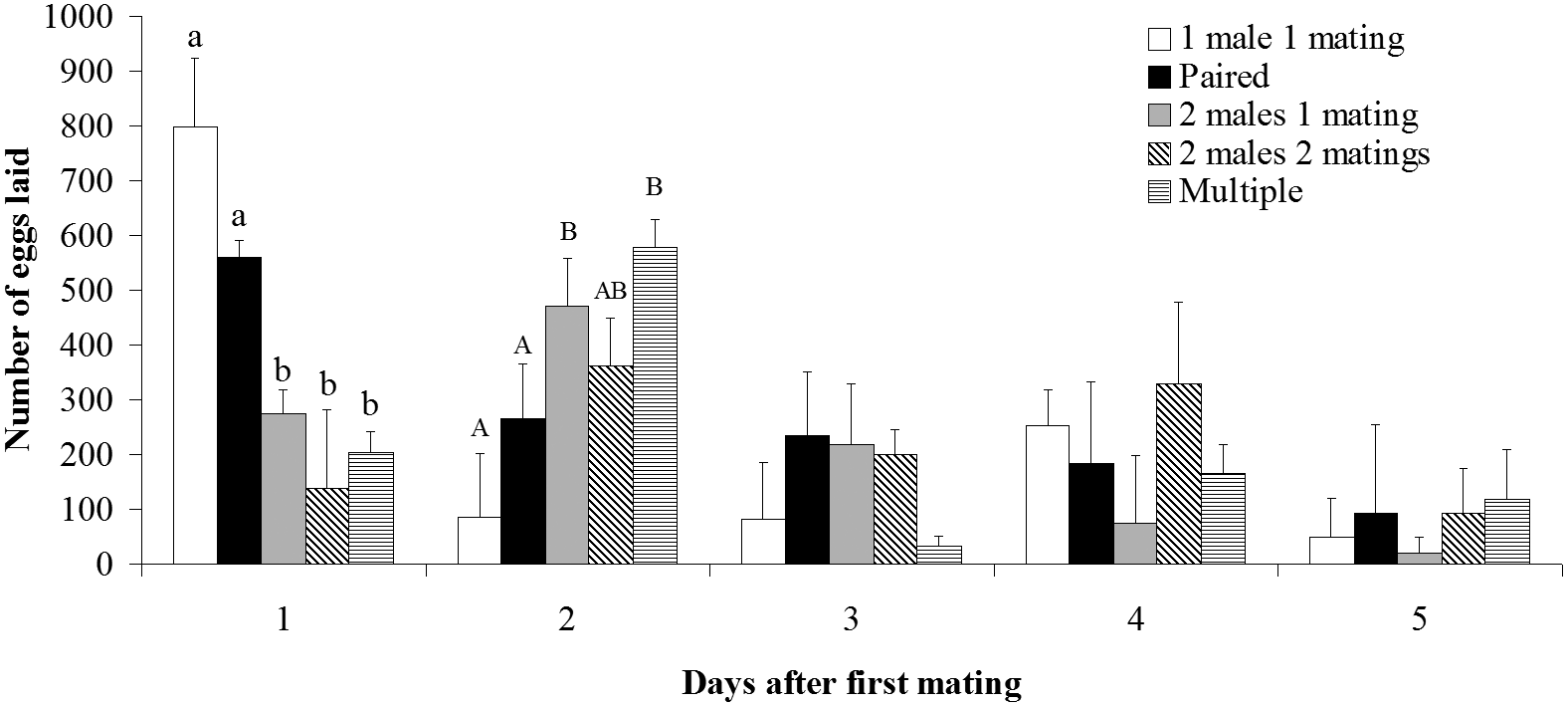
Daily oviposition patterns in relation to mating treatments in *S. litura*. Within the same oviposition night, bars with different letters are significantly different (*P*<0.05).

No significant differences were found on fecundity, fertility, fertility rate, hatched eggs, hatch rate and longevity between treatments of Paired, Multiple, 1 male 1 mating, 2 males 1 mating and 2 males 2 matings (MANOVA: *F*_24,311_ = 0.793, *P* = 0.743) (Table 1). However, significant differences were found between treatments in daily fecundity (Fig. 3). On the first oviposition night (the subsequent night after first mating), females that were caged with novel males (Multiple, 2 males 1 mating and 2 males 2 matings treatments) laid significantly fewer eggs than females caged with their previous mates (treatment Paired) and females were caged individually without males (treatment 1 male 1 mating) (ANOVA: *F*_4,56_ = 4.496, *P* = 0.003). However, on the second oviposition night, Multiple and 2 males 1 mating females laid significantly more eggs than females of Paired and 1 male 1 mating treatments (ANOVA: *F*_4,56_ = 2.872, *P* = 0.031).

**Table 1:** Effect of multiple mating on female reproductive output and longevity in *S. litura* *.

Parameter	Mean ± SE	*F*	*DF*	*P*
Fecundity (no. of eggs laid)	1176±68	0.504	4,56	0.733
Fertility (no. of fertilized eggs laid)	1098±65	0.388	4,56	0.816
Hatched eggs	971±58	0.576	4,56	0.681
Fertility rate (%)	93.9±0.73	0.820	4,56	0.518
Hatch rate (%)	88.9±1.49	1.658	4,56	0.173
Female longevity (days)	6.57±0.18	0.586	4,56	0.674

*There were no significant differences between treatments for any of these parameters.

## Discussion

Mating event data and spermatophore number in females indicate that both sexes of *S. litura* mate multiply regardless of mating with a novel or previous mate (Figs. 1 & 2). Multiple mating may benefit females through direct benefits such as sperm and/or nutrient replenishment or through reduced costs of harassment (convenience polyandry) [1], [6], [7]. In a number of species of Lepidoptera, females receiving more sperm or male investment show increased fecundity, fertility and longevity [39]–[43]. However, results of the present study do not support the sperm and nutrient replenishment hypotheses because neither the number of mates nor the number of copulations achieved by females significantly increased female fecundity, fertility and longevity in *S. litura*. Moreover, results of this study also do not support the convenience hypothesis [44]; even when females were exposed to a virgin male each night for 6 nights, they only mated approximately two times on average (Fig. 1) and did not show reduced longevity compared to females from other treatments (Table 1).

Why do females mate again when they already have sufficient sperm to fertilize all their eggs? Several genetic benefit hypotheses have been suggested to answer this question, such as the good gene [8], [9], genetic incompatibility [10]–[13] and genetic diversity hypotheses [4], [14]–[18]. These hypotheses require sperm from different males to fertilize eggs competitively [45] and/or the offspring fathered by multiple males [46]. To maximize genetic benefits from multiple mating, females from Lepidoptera [4] and other taxa [19]–[24] have evolved strategies to prefer novel versus previous mates in their subsequent matings.

In this study, we observed that females showed different oviposition patterns when caged with a novel male on the subsequent night after first mating in comparison with females caged with previous mates (Fig. 3). On the first oviposition night (the subsequent night after first mating, one-time mated females were exposed to previous or novel mates on this night), females that were caged with novel males laid significantly fewer eggs than females caged with previous mates and solitary females, suggesting delayed oviposition by females when novel mates are present. By contrast, on the second oviposition night, females that were caged with novel males laid significantly more eggs than females caged with previous mates and solitary females. A similar oviposition pattern has also been found in the hide beetle *D. maculatus* [21] and the Mediterranean flour moth *E. kuehniella* [4]. Females of *D. maculatus* do not lay eggs until they have mated several times with different males for genetic benefits [21], which may because they have a long adult lifespan (>40 days). In *S. litura*, female lifespan is short (approximately 10 days) and their best mating period is during the first few days after eclosion due to the decline of sex pheromone production [47]. This may explain why *S. litura* females chose to lay eggs quickly when no males were around or in the presence of their previous mates.

In the present study, we recorded male courtship behavior and female calling behavior in females encountering novel males and females encountering their previous mates on the subsequent night after first mating (Fig. 1). The results demonstrated females that have novel males present called early and more often than females that have previous mates present, whereas no significant differences were found in male courtship between treatments. In addition, our previous study [32] has demonstrated that males showed similar courtship patterns on the second and third night after eclosion when paired with the same female. These results suggested that females of *S. litura* can distinguish between novel and previous mates and prefer novel versus previous mates, whereas males generally like to remate with both the same and different females. However, such female preference for new mates did not result in significantly higher remating rates in females caged with new mates than females caged with previous mates (Figs. 1 & 2). This is different than the results found in other species where females mated more often with novel males than with previous mates (e.g. [4], [19]). In *S. litura*, this may be because: 1) although a normal mating is enough for females to fertilize their whole eggs (Table 1), it is better to remate several times to ensure enough sperm reserves because they may have mated with a male with a low number of sperm in the first mating (recent mated or immature male) (e.g. [48]), 2) females have a very short reproductive period [47], and 3) males generally like to remate with both the same and different females (Fig. 1b). As a consequence, females will mate again with the same male when no novel males are available.

A previous study of females mated with two males in *S. litura* has showed a last male sperm precedence pattern [49]. *S. litura* females usually mate the first time on the second night after eclosion and approximately 60% of females can mate a second time in the subsequent two days [32], [49]. Sperm need several hours (>3 h) to reach the spermatheca and become mature; thus, females usually do not lay eggs on the same night after mating [32], [49]. In the present study, in twice mated females with two males, therefore, eggs laid on the first oviposition night should be mainly fathered by the first male and the eggs laid after the first oviposition night should be mostly fathered by the second male (Fig. 2). Therefore, multiple mating in females has enhanced the progeny genetic diversity in *S. litura*. Increased genetic diversity within a brood can increase offspring fitness by reducing competition due to different genotypes better partitioning limited resources [14] and by reducing disease transfer as different genotypes may have different susceptibilities or resistance to parasites or pathogens (e.g. [16]). Although not yet tested experimentally, it is possible that enhanced genetic diversity within a brood may be a way of inbreeding avoidance if offspring live together and sibling matings occur often [18]. In *S. litura*, eggs are laid in clusters (a cluster contains 50–300 eggs) that are several layers thick and are covered with hair from the female’s abdomen, which can prevent predation of the eggs by natural enemies [50]. However, offspring competition, inbreeding and disease transfer risks are thus increased due to offspring living together in a high population density. Therefore, genetic diversity in progeny is valuable for *S. litura* and thus genetic benefits should be the main force behind the evolution of female behavioral strategies found in the present study.

The present study also found that males generally remate regardless of whether the female is a previous mate or not, which is different to previous studies where males in most species prefer novel mates [26] and in some cases prefer previous mates [28], [29]. Males’ preferring novel mates for mating will significantly improve males’ reproductive success [28], [29]. One possible explanation for males’ preferring previous mates for mating may be that males can increase their reproductive success by minimizing the opportunity for postcopulatory sexual selection [19]. Similarly, the no preference for new or previous mates in *S. litura* males may also the consequence of conflict between the sexes, in which females actively to mate with different mates to maximize the benefits from multiple mating and males seeking to minimize sperm competition [19], [49]. Further studies are needed to explore the evolutionary significance of such male behavior.
